# Novel Approach Methodologies in Modeling Complex Bioaerosol Exposure in Asthma and Allergic Rhinitis Under Climate Change

**DOI:** 10.1017/erm.2025.7

**Published:** 2025-03-12

**Authors:** Esra Atalay-Sahar, Ece Yildiz-Ozturk, Su Ozgur, Arzu Aral, Emre Dayanc, Tuncay Goksel, Ralph Meuwissen, Ozlem Yesil-Celiktas, Ozlem Goksel

**Affiliations:** 1 Translational Pulmonary Research Center (EgeSAM), Ege University, Izmir, Türkiye; 2 Department of Food Processing, Food Technology Programme, Yasar University, Izmir, Türkiye; 3 Regional Hub for Cancer Registration in Northern Africa, Central and Western Asia, WHO/IARC GICR, Izmir, Türkiye; 4 Department of Immunology, Faculty of Medicine, Yeditepe University, Istanbul, Türkiye; 5 Basic Medical Sciences, Faculty of Medicine, İzmir University of Economics, Izmir, Türkiye; 6 Koch Institute for Integrative Cancer Research at MIT, Cambridge, MA, USA; 7 Department of Pulmonary Medicine, Division of Immunology and Allergy, Faculty of Medicine, Ege University, Izmir, Türkiye; 8 Department of Bioengineering, Faculty of Engineering, Ege University, Izmir, Türkiye; 9 ODTÜ MEMS Center[CMT2], Ankara, Türkiye

**Keywords:** airway diseases, air pollution, allergy, asthma, climate change, fungus, NAMs, pollen

## Abstract

The undeniable impact of climate change and air pollution on respiratory health has led to increasing cases of asthma, allergic rhinitis and other chronic non-communicable immune-mediated upper and lower airway diseases. Natural bioaerosols, such as pollen and fungi, are essential atmospheric components undergoing significant structural and functional changes due to industrial pollution and atmospheric warming. Pollutants like particulate matter(PMx), polycyclic aromatic hydrocarbons(PAHs), nitrogen dioxide(NO_2_), sulfur dioxide(SO_2_) and carbon monoxide(CO) modify the surface and biological properties of atmospheric bioaerosols such as pollen and fungi, enhancing their allergenic potentials. As a result, sensitized individuals face heightened risks of asthma exacerbation, and these alterations likely contribute to the rise in frequency and severity of allergic diseases. NAMs, such as precision-cut lung slices(PCLS), air–liquid interface(ALI) cultures and lung-on-a-chip models, along with the integration of data from these innovative models with computational models, provide better insights into how environmental factors influence asthma and allergic diseases compared to traditional models. These systems simulate the interaction between pollutants and the respiratory system with higher precision, helping to better understand the health implications of bioaerosol exposure. Additionally, NAMs improve preclinical study outcomes by offering higher throughput, reduced costs and greater reproducibility, enhancing the translation of data into clinical applications. This review critically evaluates the potential of NAMs in researching airway diseases, with a focus on allergy and asthma. It highlights their advantages in studying the increasingly complex structures of bioaerosols under conditions of environmental pollution and climate change, while also addressing the existing gaps, challenges and limitations of these models.

## Introduction

Climate change, urbanization and rising air pollution are significantly altering the composition, behaviour, and impacts of bioaerosols, including pollen, fungal spores and other biological particles (Refs 1, 2). These atmospheric components, critical to ecological balance, are becoming increasingly potent triggers of allergic diseases due to their interactions with environmental pollutants. Such changes include earlier flowering and pollination periods, enhanced fungal sporulation, increased pollen concentrations and shifts in species composition and allergenic potential (Refs 3, 4, 5). These alterations have contributed to the rising prevalence of non-communicable, immune-mediated diseases, particularly asthma and allergic rhinitis (AR), both of which are closely linked to immune dysregulation and epithelial barrier dysfunction in the respiratory system (Refs 6, 7, 8).

Allergic rhinitis affects up to 50% of the global population and 23–30% of Europeans, while asthma affects >5% of any studied population, with a global prevalence of 9.79% in 2019 (Refs 9, 10, 11). The synergistic effects of pollutants, such as ozone (O₃), nitrogen dioxide (NO₂) and particulate matter (PM₂.₅), with bioaerosols like pollen exacerbate these diseases. For instance, long-term PM₂.₅ exposure has been linked to 30% of asthma cases globally and contributes significantly to emergency visits for asthma in regions such as India and China (Refs 12, 13). Additionally, elevated CO₂ levels have been shown to increase both pollen production and allergenic protein levels in species like sawtooth oak, further intensifying the burden of allergic diseases (Ref 14).

Pollutants directly modify the structural and biochemical properties of pollen. Oxidative and nitrative stress caused by O₃ and NO₂ alters protein conformation, leading to increased allergenicity. For example, the major birch pollen allergen Bet v 1 becomes nitrated under polluted conditions, significantly enhancing its IgE-binding capacity and allergenic potential (Refs 15, 16). Similarly, the allergen Pla a3 from Platanus pollen undergoes structural changes through nitration and oxidation upon NO₂ and O₃ exposure, resulting in increased atmospheric release, immunogenicity and stability. These changes not only heighten allergic responses but also worsen respiratory conditions like pollen-induced pneumonia (Ref 17).

**Figure 1.**
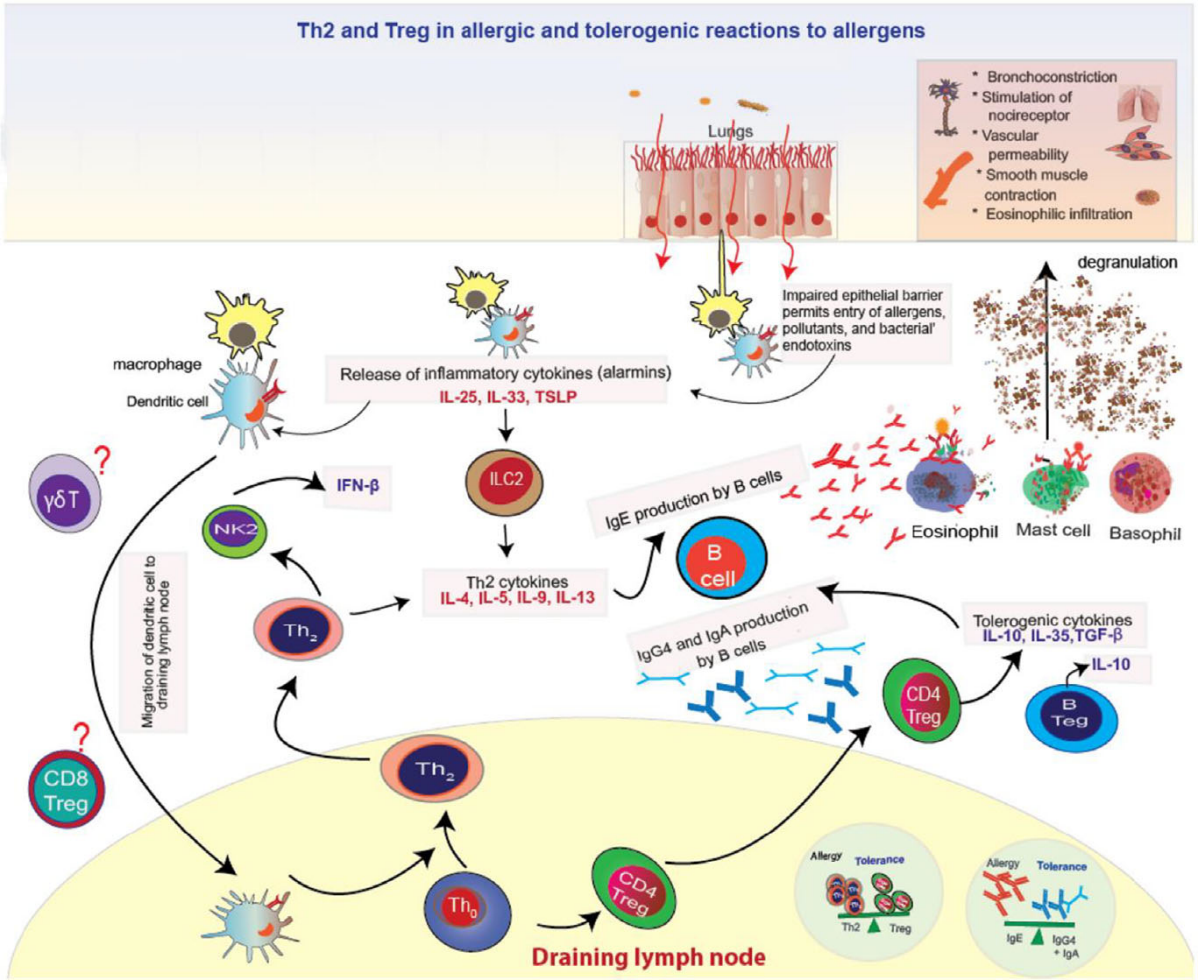
Allergic reactions on encountering allergens or pollutants (reproduced from Ref 22 under the terms of the Creative Commons Attribution CC BY 4.0 License).

The respiratory epithelial barrier, a multilayered defence system, is particularly vulnerable to pollutant-mediated damage. Tight junction proteins such as ZO-1, occludin and claudin-4 are disrupted by oxidative stress, leading to increased permeability and facilitating the penetration of allergens and pollutants (Ref 18). This dysfunction triggers the release of epithelial-derived cytokines such as IL-25, IL-33 and TSLP, which activate type 2 innate lymphoid cells (ILC2s) and naïve T cells, promoting Th2 cytokine production (IL-4, IL-5, IL-9 and IL-13) and class switching to IgE in B cells (Refs 19, 20). IgE binds to FcεRI on mast cells, basophils and eosinophils, inducing the release of histamine, leukotrienes and prostaglandins, which contribute to the hallmark symptoms of allergic rhinitis and asthma, including sneezing, nasal congestion, chronic throat irritation, cough and shortness of breath (Figure 1) (Refs 21, 22).

The interplay between environmental stressors and immune mechanisms complicates the clinical phenotypes of allergic diseases. Pollutant-induced oxidative stress enhances airway hyperreactivity and disrupts immune regulation. For instance, pollutants reduce Th1 and Th17 cytokine production, impairing bacterial clearance and increasing susceptibility to infections (Ref 23). Concurrently, overactivation of neutrophilic responses in models combining pollutant and bacterial exposures has been linked to severe tissue damage (Ref 24). These effects underline the complexity of immune dysregulation in the context of environmental changes.

The interaction between pollutants and bioaerosols also has implications for epithelial barrier integrity and immune responses during critical developmental periods. Augmented exposure to residential air pollutants has been associated with increased rates of eczema and aeroallergen sensitization in children, while systematic reviews highlight the impact of pollutants on allergic disease development during perinatal periods (Refs 4, 15, 25, 26). The increasing prevalence of allergic airway diseases, projected to affect four billion individuals by 2050, underscores the urgent need to better understand the mechanisms driving these trends (Ref 27).

Laboratory and field studies have provided critical insights into these processes. Proteins in pollen exposed to polluted urban air or gas mixtures of NO₂ and O₃ are efficiently oxidized, nitrated or oligomerized, significantly altering their biochemical properties and allergenic potential (Refs 28, 29). However, traditional models are often insufficient to capture the complexity of these interactions, highlighting the need for advanced approaches.

Novel Approach Methodologies (NAMs) offer a promising avenue for investigating the multifactorial mechanisms underlying these interactions. NAMs include advanced in vitro, in silico and exposure simulation models capable of replicating the dynamic interplay between pollutants and bioaerosols (Ref 30). These methodologies not only enhance our understanding of disease pathogenesis but also support the development of new diagnostics, therapeutic interventions and preventive strategies.

Such models can significantly enhance the decision-making process in a variety of clinical contexts related to drug administration. Furthermore, they possess the capability to be scaled up for high-throughput applications and can be easily employed in formulation stages. Such models can help inform decisions about different clinical contexts for drug administration and the efficacy of new treatments. Piqué and De Servi, 2018, evaluated the effect of Rhinosectan® spray, a medical device containing xyloglucan, on the function of the nasal ciliary using the air–liquid interface (ALI). They demonstrated that the application of Rhinosectan® in vitro model does not impair ciliary movement, enhances phagocytic capacity, reduction mucin secretion, which are optimal properties for use in the management of rhinitis and associated conditions (Ref 31)

This review aims to examine NAMs, highlighting their advantages and limitations in modeling chronic inflammatory airway diseases, with a specific focus on asthma and allergic rhinitis, which are the diseases most closely associated with the inhalation of bioaerosols altered by environmental pollution, within the context of climate change. By addressing the dynamic interplay between environmental pollutants, pollen, fungi and other bioaerosols, we aim to provide insights into advanced experimental and computational models that can unravel the multifaceted mechanisms underlying airway barrier dysfunction and immune dysregulation, with the ultimate goal of informing targeted therapeutic and preventive strategies.

## Human tissue-based *ex vivo* and *in vitro* allergy and asthma models

### *Ex vivo* cultures

Precision-cut lung slices (PCLS) are extensively utilized in asthma and allergic disease research due to their ability to replicate physiologically relevant interactions within lung tissue. However, their application in bioaerosol studies remains limited, representing a significant gap in the field. PCLS models have the potential to simulate real-time interactions between lung tissue and bioaerosols, as demonstrated in studies investigating airway contractility. A notable example is the study by Bai et al. (Ref 32), which explored the relationship between cholinergic stimulation and airway smooth muscle contractility using PCLS derived from young and adult donor lungs treated with methacholine. Their findings revealed that airway smooth muscle undergoes age-dependent changes during postnatal lung development, influencing contractile phenotype and airway hyperresponsiveness. Moreover, CD38 was identified as a critical mediator of cholinergic dysregulation in young children, emphasizing the ability of PCLS to model age-specific mechanisms and real-time physiological responses in asthma. Airway sensitivity and reactivity to direct (e.g., histamine, methacholine) and indirect (e.g., exercise, cold air, hyperventilation) challenges are hallmarks of asthma, correlating airway smooth muscle function with disease severity. In their 2023 review article titled “Advances in Respiratory Physiology in Mouse Models of Experimental Asthma,” Caroll et al. stated that PCLS models uniquely replicate the interplay between airway smooth muscle and the surrounding parenchyma, providing an accurate platform to evaluate these dynamics. (Ref 33).

An illustrative example of incorporating bioaerosols into PCLS ex vivo models is provided by Redes et al. (Ref 34). This study examined the impact of the serine protease Alp1 allergen from *Aspergillus fumigatus* on airway contractility. Exposure to Alp1 was shown to enhance airway contractility in murine PCLS models, and further experiments with human airway smooth muscle cells confirmed that Alp1 directly increased contractile force. These findings highlight the utility of PCLS in simulating bioaerosol interactions under controlled and physiologically relevant conditions, making them a valuable tool for studying pollutant-induced respiratory effects.

Asthma, a heterogeneous inflammatory disorder of the airways, is characterized by symptoms such as airway inflammation, hypersensitivity, bronchoconstriction and airflow obstruction. It is broadly categorized into type 2 (T2) and non-T2 asthma, depending on the presence or absence of a T2 immune response. T2 asthma is associated with an increased susceptibility to allergens, which triggers immune responses involving eosinophil recruitment and the production of cytokines such as IL-4, IL-5 and IL-13. PCLS models derived from asthmatic patients closely mimic these physiological responses, including bronchoconstriction, inflammation and airway hyperresponsiveness following exposure, thereby reflecting the pathophysiological features observed in real-life asthma patients (Ref 35). PCLS models effectively capture the interplay between genetic and environmental factors, as demonstrated by the expression of genes involved in both T2 and non-T2 asthma pathogenesis, along with cytokines associated with these phenotypes, such as IL-25, TSLP, the TTP family and IL-13, in lung sections from asthmatic patients (Refs 36, 37, 38). To evaluate the therapeutic potential of targeting these asthma-related genes, a siRNA delivery system was developed and tested in a PCLS inflammation model by Kandil et al. This system comprised polyethyleneimine (PEI) as a polycationic carrier, transferrin (Tf) as a targeting ligand and melittin (Mel) as an endosomal escape agent. This PEI-based system, which can be nebulized for direct lung delivery, demonstrated therapeutic efficacy by reducing the inflammatory cascade through targeted GATA3 knockdown in activated T cells (Ref 39).

**Figure 2.**
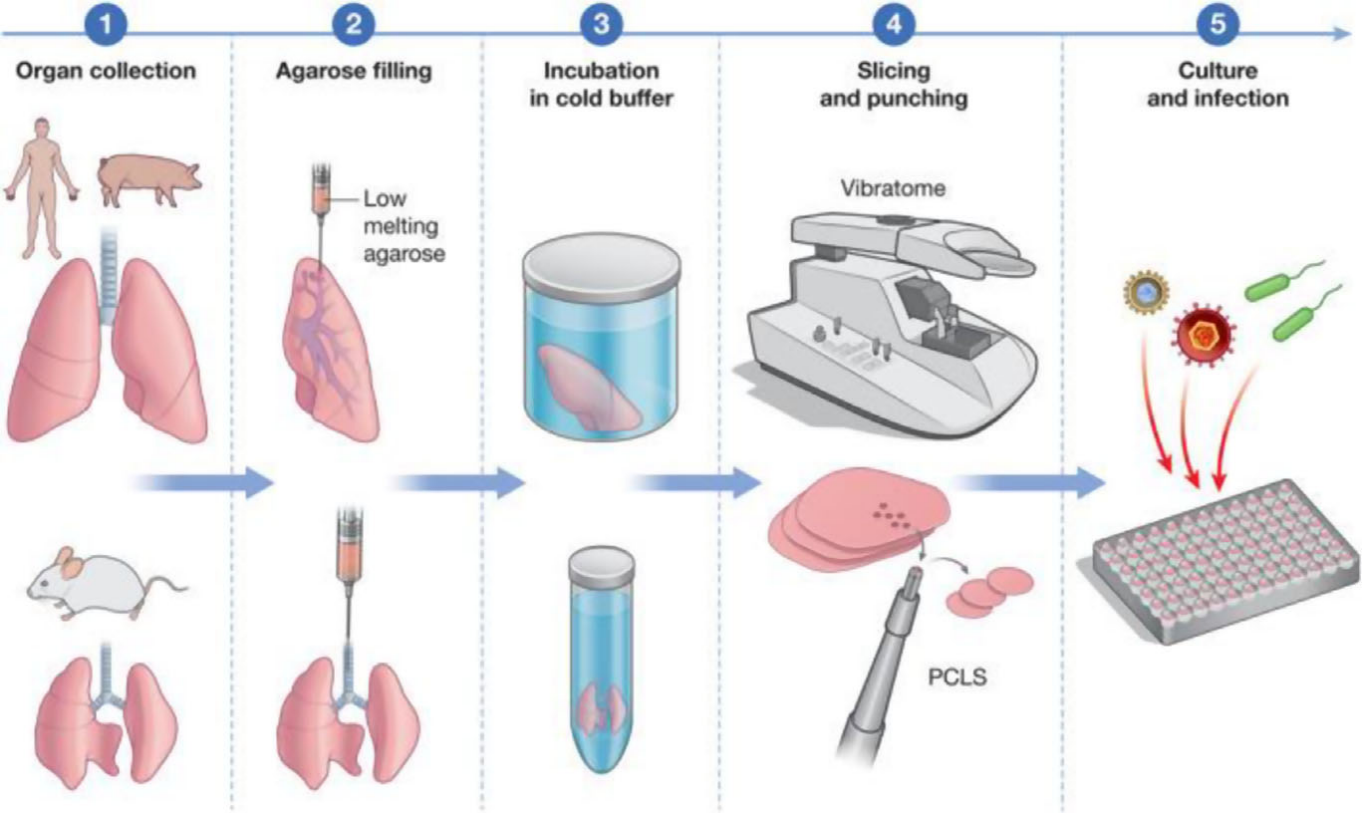
Generation of precision‐cut lung slices (PCLS) (adapted with open access permission under a Creative Commons Attribution license from Ref 40).

In their 2023 study, Deniz et al. demonstrated the potential of 3D bioprinting technologies in reconstructing human nasal epithelial models and successfully created a model mimicking the complex three-dimensional structure of the natural nasal epithelium using progenitor human nasal epithelial cells (hNECs). They also highlighted that advances in tissue slicer technology have enabled the rapid and reproducible preparation of PCLS, facilitating high-throughput analyses and detailed investigations for various applications such as infection studies, disease modeling and drug screening (Figure 2) (Ref 41).

Unlike traditional 3D lung tissue culture models that rely on single-cell lines, PCLS preserves multiple cell types and maintains the functional cellular relationships within the tissue. Morphologically, PCLS retain critical components of lung structure, including small airways, respiratory parenchyma, immune cells and connective tissue (Ref 42). These features make PCLS particularly suitable for studying chronic inflammatory diseases such as asthma and allergic rhinitis. The ability to maintain the structural integrity of lung tissue is especially critical for replicating complex immune responses, including bronchoconstriction and airway hyperresponsiveness (Refs 43, 44).

In summary, there is still much to be explored regarding the integration of PCLS into asthma and allergic disease research under dynamic climate and weather conditions. By preserving tissue architecture and enabling real-time analysis of lung tissue interactions, PCLS offer significant advantages over traditional in vitro systems, serving as a powerful tool for investigating pollutant-induced respiratory pathology, airway dynamics and immune responses; expanding their application to bioaerosol research will further enhance our understanding of these interactions and improve the precision of respiratory research.

### Air–liquid interface (ALI) based in vitro culture models

**Figure 3.**
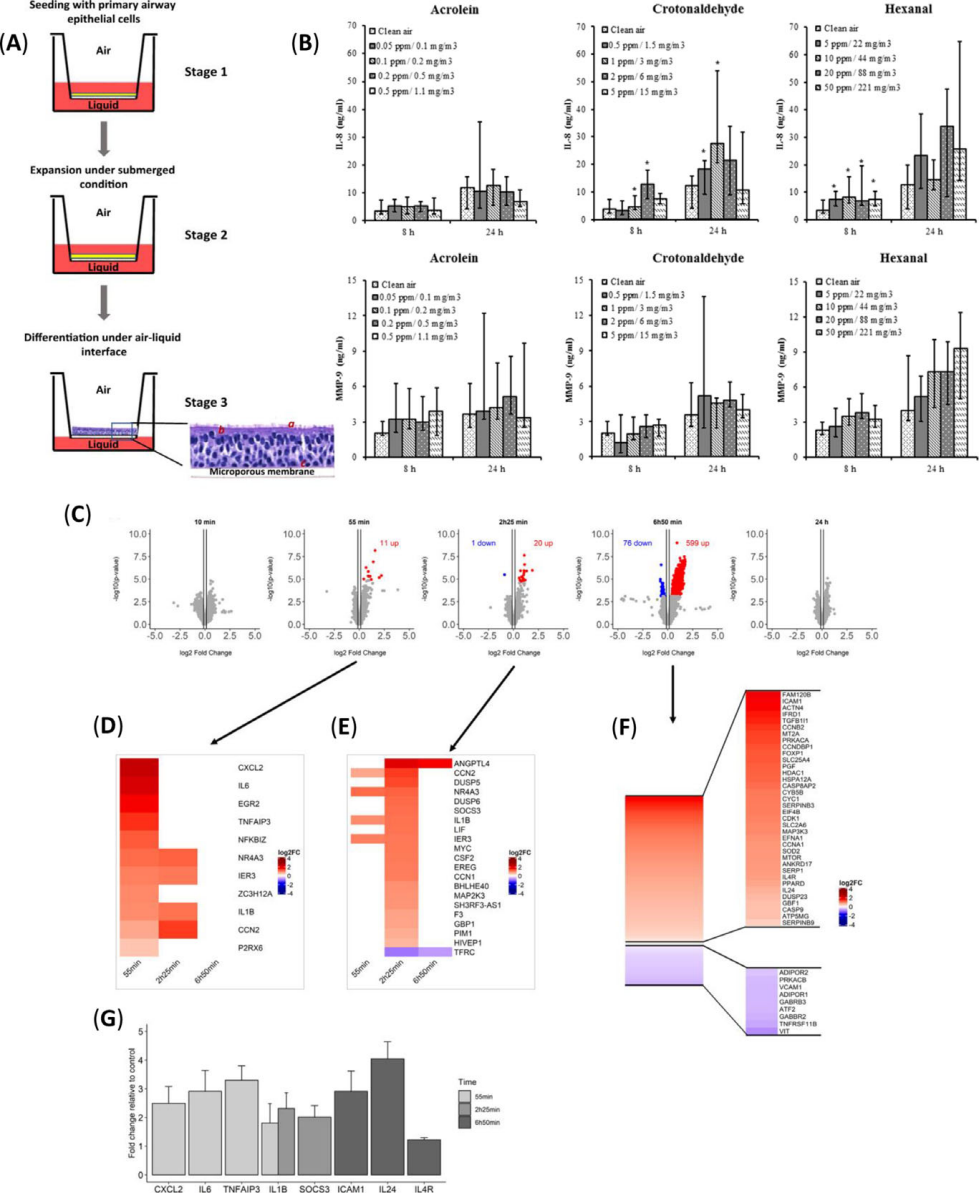
**A.** The schematic diagram of the ALI airway model (adapted with permission from Ref 46, Copyright 2020 The Society for in vitro Biology). **B.** Interleukin-8 (IL-8) and matrix metalloprotein-9 (MMP-9) secretion in basal media after exposure of primary bronchial epithelial cells at the air–liquid interface to 0.05–0.5 ppm (0.1–1.1 mg/m3) acrolein, 0.5–5 ppm (1.5–15.0 mg/m3) crotonaldehyde, and 5–50 ppm (22–221 mg/m3) hexanal for 30 min (Reproduced from Ref. 50 under the terms of the Creative Commons Attribution CC BY 4.0 Licens). **C.** Transcriptomic analysis of the effect of 10 mg whole birch pollen on immortalized human bronchial epithelial BEAS-2B cells, at the air–liquid interface. Volcano plots and (adapted with open access permission under a Creative Commons Attribution License Ref. 51). **D, E.** Heatmaps of all significantly regulated genes for the 55 min and 2 h 25 min incubation times, respectively (adapted with open access permission under a Creative Commons Attribution license from Ref 51). **F.** Heatmap of all the significant regulated genes for 6 h 50 min incubation time, with focus on the most relevant genes. (adapted with open access permission under a Creative Commons Attribution license from Ref 51). **G.** mRNA expression of selected genes by qRT-PCR that were expressed in transcriptome data (adapted with open access permission under a Creative Commons Attribution license from Ref 51).

The use of transwell inserts as differentiation platforms has gained increasing popularity in developing lung in vitro models that enable respiratory epithelial cells to be cultured at an “air–liquid interface” (ALI), simulating a more realistic airway environment (Ref 45). ALI culture is a well-characterized example of organoid culture. In this model, the transwell membrane divides the culture environment into two compartments, where the basal surface of the cells is in contact with the culture medium, and the apical surface is exposed to air, promoting cellular differentiation (Figure 3A) (Ref 46). Under these conditions, primary airway cells differentiate into basal, ciliated and goblet cells, replicating the structural and functional characteristics of human airway epithelium (Ref 47).

ALI models are widely used to mimic respiratory diseases, including chronic obstructive pulmonary disease (COPD), asthma, cystic fibrosis, lung cancer and respiratory infections. They are also utilized to assess the impact of inhaled substances, such as pollutants or therapeutic aerosols, on lung health (Ref 48). Moreover, ALI culture serves as an important in vitro methodology for studying the asthmatic epithelium with high similarity to in vivo conditions. ALI cultures using primary human bronchial epithelial cells derived from asthmatic patients have been employed to detect and classify novel patient subsets, such as IL-6TS–driven subsets, identified through epithelial IL-6TS-specific gene signatures (Ref 49). Another study cultured primary basal human bronchial epithelial cells under ALI conditions with fibroblasts to investigate the inflammatory and oxidative stress effects of aldehydes present in cigarette smoke (Figure 3B) (Ref 50). Candeias et al. (Ref 51) developed a system for exposing human bronchial epithelial cells to whole, real-life pollen grains in ALI conditions and analyzed their effects. The system developed by the researchers enabled cells to be dosed more gently and reliably, replicating exposure in a way that reflects how humans experience it in real life (Figure 3C–G) (Ref. 51).

In their 2020 study titled Biological effects of allergen–nanoparticle conjugates: uptake and immune effects determined on hAELVi cells under submerged versus air–liquid interface conditions, Mills-Goodlet et al. reported that, under ALI conditions, the thin liquid layer on top of the cells reduces issues such as nanoparticle agglomeration, diffusion, sedimentation and dissolution compared to submerged culture (Ref 52). In another study, Zimmermann E.J. et al. (Ref 53) evaluated the toxic effects of anthropogenic pollutants, birch pollen extract (BPE), and house dust mite extracts on bronchial epithelial cells under ALI culture conditions. The ALI culture system demonstrated its strength by facilitating a more rapid and prominent regulation of pro-inflammatory and xenobiotic signals in response to allergen exposure (Ref 53). Most studies to date have focused on pollen-cell interactions at ALI conditions to explore cellular mechanisms in the bronchial epithelium. However, some studies have used well-differentiated primary human bronchial epithelial cells or human nasal epithelial cells grown in ALI conditions to investigate transcriptomic, proteomic, cellular, molecular and immunological responses to widespread fungi such as *A. fumigatus* (Refs 54, 55, 56). Gilles et al. (Ref 57) took a step further by integrating data from human exposure cohorts, mouse models and ALI cultures to evaluate the potential modulation of antiviral immunity by pollen exposure. Their findings revealed that pollen exposure alters antiviral defense mechanisms in the respiratory epithelium and these in vitro NAM results were successfully validated in vivo (Ref 57).

Asthma research has greatly benefited from the ALI culture of bronchial epithelial cells. As a representative of NAMs, ALI cultures excel in high-throughput screening of the effects of complex bioaerosols on the respiratory system. These cultures allow researchers to observe the combined impacts of pollen or fungal spores on airway inflammation, mucus production and bronchoconstriction, representing a significant improvement over traditional cell cultures that fail to capture such synergistic effects. These characteristics make ALI models highly suitable for studying specific airway disease phenotypes and responses to environmental stimuli, including pollutants and bioaerosols. (Refs 58, 59).

In summary, ALI cultures, unlike conventional, submerged systems, allow direct exposure of the apical surface to airborne particles, providing a dynamic platform for real-time assessment of bioaerosol interactions with epithelial cells. This advanced capability is essential for accurately modeling chronic airway diseases, which are especially susceptible to the effects of environmental pollution and climate change.

### Lung organoids

Human alveolar an airway organoids derived from human pluripotent stem cells, embryonic progenitor cells and adult lung tissues—both healthy and diseased—have been utilized as NAMs to model various cellular and molecular characteristics of the alveolar epithelium (Refs 60, 61).

Lee et al. (Ref 62) compared the transcriptomes of organoids derived from lung tissues of healthy individuals and patients with the chronic respiratory disease by incorporating in vitro datasets from the Human Lung Cell Atlas. They demonstrated that, despite differences in the proportions of cellular composition, cellular diversity and transcriptional cell states are significantly maintained in all in vitro models and are comparable to those of in vivo human lungs (Ref 62). Another study by Vazquez-Armendariz et al. (Ref 63) established a three-dimensional (3D) murine bronchioalveolar lung organoid (BALO) model that enables clonal expansion and self-organization when co-cultured with lung-resident mesenchymal cells (Ref 63). Such models contribute to emerging in vitro (and ex vivo) approaches for studying pulmonary development and diseases. In a different study, they established a human-induced pluripotent stem cell-derived alveolar epithelial cell-based organoid culture system for the alveolus. They found that alveolar epithelial cell-based organoids in 3D culture were suitable for assessing the cytotoxicity of chemical substances (Ref 64).

Alveolar organoids are used for studying immune cell presentation for modeling inflammatory pulmonary diseases. However, their application in the bioaerosol area, particularly in research on chronic airway diseases and allergies, remains limited. Organ-on-a-chip platforms and other NAMs, such as ex vivo systems, PCLS and ALI, can enhance organoid-based studies by providing greater cellular and structural complexity, thereby serving as valuable models for human lung diseases. (Ref 60).

Human stem cells or primary cells, which are isolated from surgically excised human tissue or non-transplantable organs, are predominantly utilized in 3D models. These cells can be cultured at the air–liquid interface or submerged, mimicking in vivo-like tissue conditions and their mature, differentiated tissue state. However, these models have inherent limitations, including a lack of cryopreservability, reproducibility and limited cell diversity. Unlike stem cell-based models, they are not self-renewing. Organoids, derived from tissue-specific adult stem cells (ASCs) or induced pluripotent stem cells (iPSCs), overcome some of these limitations. Human ASC-derived organoids, in particular, are advantageous because they can be directly generated from patient tissue, enabling personalized medicine approaches by replicating disease phenotypes and environmental interactions specific to individual patients. However, their application remains constrained by challenges in tissue accessibility and the requirement for prior knowledge of tissue culture conditions (Ref 65).

In summary; as highlighted in the 2024 review by Purev et al. (Ref 66) titled Alveolar Organoids in Lung Disease Modeling, lung organoids have been developed using two main systems: air–liquid interface (ALI) culture on inserts exposed to air and submerged culture without air exposure. Particularly for airway disease modeling associated with bioaerosol exposure, organoid ALI cultures stand out as valuable NAMs. Yet, despite their potential, lung organoids still have some limitations; such as the lack of endothelial and immune cell co-culture conditions, native extracellular matrix (ECM) components and physiological-like mechanical stress. While spheroidal models overcome some drawbacks of 2D monocultures, they still lack an external ALI for cilia and mucus analysis. Furthermore, ethical concerns limit the use of human challenge models. Overall, lung organoids offer a promising platform for studying bioaerosol pathobiology, airway disease development and in vitro drug screening.

### Lung-on-a-chip models

**Figure 4.**
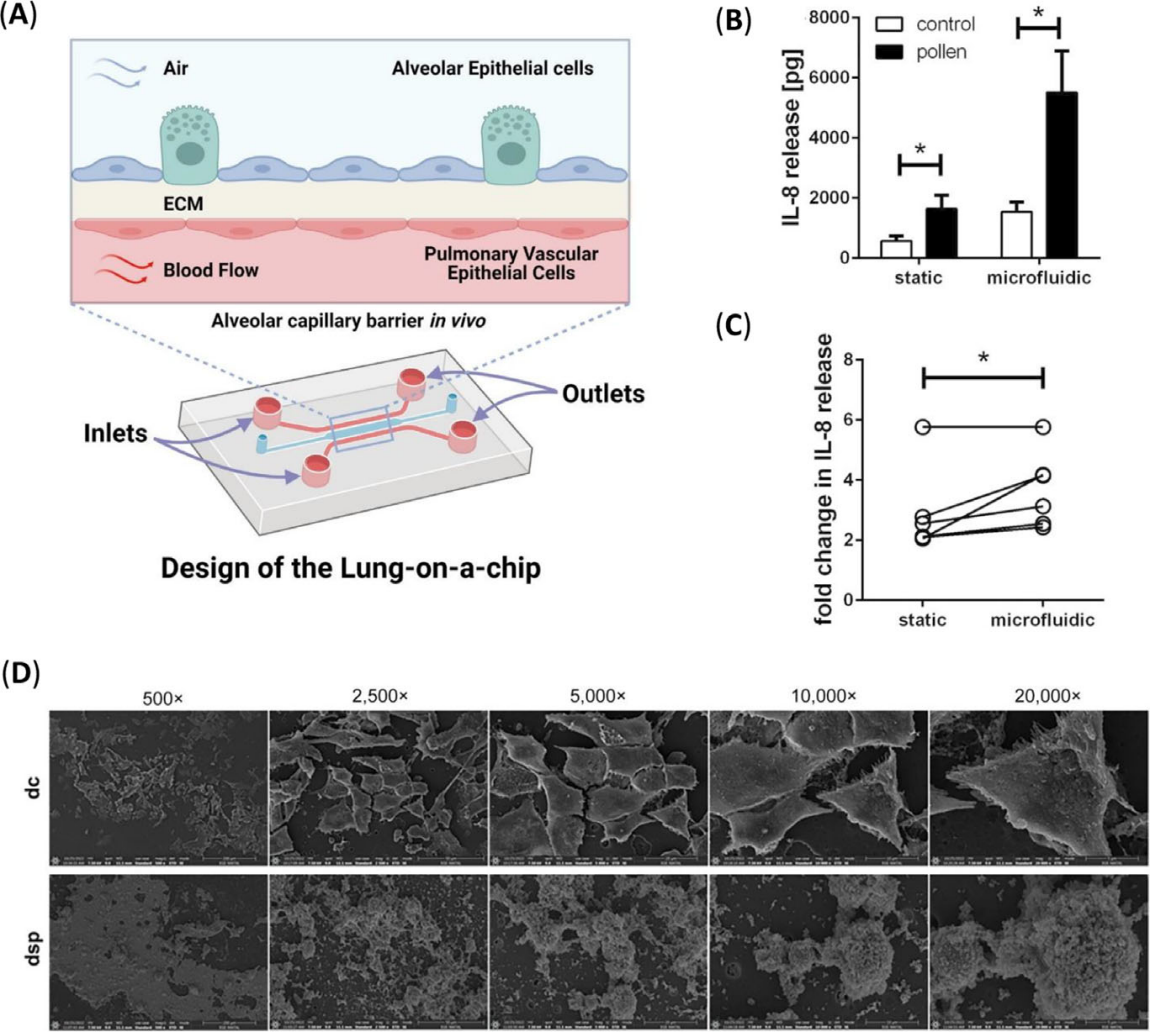
The alveolar-capillary barrier in lung-on-a-chip systems. **A.** The microfluidic lung-on-a-chip model with two different channels separated by a thin, porous membrane. Human alveolar epithelial cells and human pulmonary microvascular endothelial cells are cultured at the top and bottom of the extracellular matrix (ECM)-coated membrane, respectively. Once the confluency is achieved, the media from the upper channel is aspirated to culture the alveolar cells at an air–liquid interface, whereas a syringe pump is connected to the lower channel to continuously infuse media. Reproduced from Ref 67 under the terms of the Creative Commons Attribution CC BY 4.0 License. **B.** Release of IL–8 by differentiated PBECs in microfluidic compared to static culture conditions for the 24 h after pollen treatment. (adapted with open access permission under a Creative Commons Attribution license from Ref 81). **C.** Comparison of pollen-induced IL–8 release in static and microfluidic culture conditions. The x-fold change in pollen-induced IL–8 release compared to untreated control is shown (adapted with open access permission under a Creative Commons Attribution license from Ref 81). **D.** PM2.5 exposure under dynamic conditions in the on-chip platform disrupts human airway epithelial barrier integrity. SEM analysis of Calu-3 cells on the airway epithelial barrier-on-a-chip platform after 72 h of PM2.5 exposure (dc: dynamic control, DSP: dynamic silica particles, sc: static control, ssp: static silica particles conditions) (adapted with open access permission under a Creative Commons Attribution-NonCommercial license from Ref 84).

The primary advantages of lung-on-a-chip models include mimicking lung airway morphology and anatomy, involving the influence of mechanical strain due to breathing, applying flow physiology providing a specific physiological microenvironment (shear stress, viscosity and local pressures) and recapitulating *in vivo* lung tissue with the presence of constant circulation of fluids that supply and remove system components such as nutrients, metabolic wastes, hormones, cytokines and so forth within lung tissue microenvironment (Figure 4A) (Refs 68, 69). These combined advantages of microfluidic-based *in vitro* models may be attractive in advanced research steps, especially through a closer mimicry of the *in situ* physiological environment (Ref 45). Organ-on-a-chip platforms enable controlled bioperfusion through microfluidic setups, using in vitro peristaltic pumps or gravity-driven devices to achieve various flow types, thereby mimicking interactions between cells, the extracellular matrix (ECM) and atmospheric pollutants (Refs 70, 71). They provided crucial knowledge of not only general chronic respiratory diseases but also lung cancer (Refs 72, 73), asthma (Refs 74, 75), chronic obstructive pulmonary disease (COPD) (Ref 76), pulmonary fibrosis (Ref 77) and cystic fibrosis (Ref 78, 79, 80).

Blume et al. (Ref 81) developed a 3D dynamic microfluidic culture system of differentiated primary human airway epithelial cells at the air–liquid interface, designed to investigate cellular responses to environmental agents such as pollen. This model, which demonstrated increased IL-8 release upon grass pollen extract stimulation, offers high sensitivity for toxicological and pharmacological studies in chronic lung diseases (Figure 4B,C) (Ref 81). Chandorkar et al. (Ref 82) developed another in vitro 3D lung/immune cell model comprising human bronchial or small airway epithelial cells grown under ALI and perfusion conditions to explore interactions between airborne pathogens, such as *A. fumigatus*, and respiratory epithelial and immune cells. Their findings revealed that these conditions enhanced ciliogenesis, the formation of an intact mucociliary layer and epithelial barrier function, enabling advanced co-culture applications for studying airborne challenges (Ref 82). Although we do not address viral bioaerosols in this review, Nawroth et al. (Ref 83) developed an airway lung-on-chip model to study viral-induced asthma exacerbations, demonstrating altered secretion of IL-6, IFN-λ1 and CXCL10 following IL-13 treatment and evaluating neutrophil migration and immunomodulatory therapy under asthmatic and healthy conditions (Ref 83).

A recent study by our group investigated the effects of PM2.5 exposure under extreme weather conditions influenced by climate change, using a human airway epithelial barrier-on-a-chip and a human immune cell-containing bronchoscopy-derived ex vivo airway tissue slice to assess dose-dependent impacts on lung epithelial cells. PM2.5 exposure significantly disrupted the airway epithelial barrier, as evidenced by increased permeability, reduced expression of cell adhesion and barrier markers (ZO-1, Vinculin, ACE2, CD31), impaired cell viability, and elevated levels of pro-inflammatory markers (IFNs, IL-6, IL-1 s, TNF-α, CD68, CD80, iNOS), particularly under dynamic conditions. The lab-on-a-chip model, a leading NAM representative, proved as effective as PCLS in demonstrating these effects (Figure 4D). (Ref 84).

In recent years, efforts to develop and commercialize in vitro alternatives for toxicological risk assessment have yielded promising 2D and 3D cell culture models. Incorporating respiratory cells and bioactive matrix components into these models advances the understanding of physiological mechanisms, disease pathologies and drug development (Ref 85). Park et al. (Ref 86) also developed an allergen-induced asthma model by integrating a vascular platform with an airway-on-a-chip using 3D-printed cell-laden dECM bioinks, effectively recreating the interface between the airway epithelium and the vascular network (Ref 86). This model simulated respiratory symptoms, including asthmatic airway inflammation and allergen-induced exacerbation and demonstrated pathophysiologically relevant immune responses, making it a promising alternative to animal models for preclinical drug testing.

Rigorous validation and refinement of complex lab-on-a-chip models are essential to ensure their applicability in studying the effects of bioaerosols, pollutants and environmental factors on chronic airway diseases. By addressing challenges such as physiologically relevant microenvironments, standardized protocols and regulatory acceptance, these systems can advance personalized medicine. With their ability to replicate patient-specific disease phenotypes and environmental interactions, lab-on-a-chip technologies hold immense potential as transformative tools in preclinical and clinical research.

## Complementary characteristics of NAMs to animal models

The above-described advances in the use and development of in vitro techniques for studying airway diseases are quite encouraging given the necessity in preclinical research for better translatability into clinical practice. These in vitro techniques, defined here as NAMs, hold the potential to significantly increase preclinical model predictability in drug discovery (Ref 87). Does this then imply that NAMs will be far superior to current main preclinical models, such as animal disease models, or even make them completely obsolete? There is not an easy answer or maybe this question is not relevant if our main goal is to improve the quality and predictability of preclinical methods as a bridge between basic and translational research. A comparison between NAMs and animal models can help us to make better use of the strength of both systems for the improvement of our preclinical research. Arguments for the use of animal models remain strong and its application remains critical for biomedical research. Mammalian animals are very similar to humans. Mice and men share about 98% of their functional genes, only 1% less than chimpanzees and men. This implies that mice and men develop in a similar way from sperm and egg with the same kind of organs and similar reproductive, digestive, circulatory, hormonal, nervous and immune systems. Furthermore, animals are susceptible to many of the same health problems as humans – cancer, diabetes, heart and airway diseases and so forth Besides, with a shorter life cycle than humans, animal models can be studied throughout their whole life span and across several generations, a critical element in understanding how a disease processes and how it interacts with a whole, living biological system. But we should not underestimate physiological dissimilarities between mammalian species. They are real and can influence the usefulness of animal models in a significant way. For example, the human and rodent lungs differ both in gross anatomy and histology (Ref 88). Rodents have monopodial airway branching, whereas humans have dichotomous branching. Rodents lack any cartilage in their intrapulmonary airways, and they have no well-developed respiratory bronchioles. Besides, the organization of the normal airway mucus system differs in small experimental animals, such as rodents, from that in humans through lack of submucosal glands in the proximal airways. On the other hand, NAMs and especially those presented here do have an increasing physiological similarity to human pulmonary epithelial tissues and of course, are not hampered by any interspecies differences. The latter does not however imply that NAMs universally produce more predictive results compared to animal models. For instance, pathophysiological differences between pulmonary diseases such as lung cancer, asthma or COPD are obvious and necessitate a pragmatic approach for using either NAMs or animal models in drug discovery (Ref 89). Current preclinical research is rather inefficient in preventing high drug attrition rates in clinical studies. This is largely due to the highly complex and challenging nature of drug development in which understanding how therapeutic molecules and their targets interact within a multifaceted disease biology is of key importance. Consequently, this demands a research approach that combines various sophisticated tools and methods.

One critical tool in drug development research is genetically engineered mouse models **(GEMMs).** Many different knock-out and knock-in mice have shown their value in answering questions on basic biology, but these same mice can be used to model diseases for drug efficacy studies, analyze on-target and off-target therapeutic effects as well as many other biological and pharmacological questions (Ref 90). However as argued above, a critical application of these models requires a thorough understanding of their strengths and limitations concerning the background of the biological context in which they are being used as well as the hypotheses they try to solve. Without good-quality data, it will not be possible to determine whether a preclinical research method is a valid paradigm for drug development. However advanced and sophisticated GEMMs might be, they should not be used alone but always together with separate other biochemical, in vitro (NAMs), and clinical platforms generating combined data to enable our better understanding of the drug, target and disease biology (Ref 91). The need for improved preclinical research led to two separate movements in the current scientific world: one to “improve animal research” and the other to “promote NAMs.” Selective preference for each of the movements is not easy since either one of them has its advantages and disadvantages. Growing evidence showed that animal studies are often conducted and reported in such a poor way that conclusions might or should not be drawn from them (Ref 92). But then serious efforts are underway to improve the quality of animal models. At the same time, NAMs are starting to be validated as new, human biology-based approaches in drug discovery. Such NAMs as organoids and organs-on-a-chip are already being used very efficiently for high throughput drug screens and are expected to revolutionize drug discovery (Ref 93). The first movement to “improve animal research” faces its main problems in species differences which could render the extrapolation of findings from animals to humans unreliable even if performed under Good Laboratory Practice regulations (Ref 94). Highly refined GEMMs led to a significant improvement in understanding the basic biology of many diseases and therefore improved the quality of preclinical research.

NAMs also have their pitfalls and potential problems. Often transformed modified cell lines are used in some of the in vitro assays, whereas primary human cells should have been preferred. Furthermore, there is a great need for standardized cell culture conditions, obtaining data on ADME (absorption, distribution, metabolism and excretion) during drug testing. Also, data interpretation and their extrapolation from in vitro to in vivo, need serious attention (Ref 95). Both advanced animal models and NAMs are striving to reduce species differences to their respective predictive power for human clinical practice. It is obvious however that NAMs have an advantage here and harbour a great promise for more successful drug discovery. One should not forget the different impact animal models and NAMs have on the ethical issues of preclinical research. Animal models can be highly controversial although the described advanced animal models greatly reduce the numbers and suffering of animals needed to produce significant predictive results. Still, NAMs should have fewer ethical issues and mainly focus on the use of human or patient-specific tissues. Lastly, there is the practical aspect: the amount of time each different model needs to produce translational results. In general, NAMs should be faster and more cost-efficient than animal models. Overall, selective complementary use of both NAMs and significantly refined animal models should be applied for a more efficient translation of preclinical research results into clinical practice.

Are NAMs with their absence of any species difference than in the way of making animal models completely obsolete? No, not necessarily. New, even more refined, humanized animal models continue to be developed in which drugs and therapies can be tested in a completely intact in vivo system. Some of the most extraordinary strengths of NAMs are their suitability for high throughput drug screening, their functionality and their applicability in evaluating therapeutic efficacy and regulatory safety of drugs and chemicals (Ref 96)). The latter application would replace many of the “classical” animal models-based toxicology assays that already have been proven to be without high predictive power anyway (Ref 97).

## NAMs integration with computational models

NAMs, such as PCLS, ALI cultures, lung-on-a-chip models and computational modeling, serve as powerful tools for simulating complex interactions within human lung airways. When integrated with computational modeling, which incorporates data from genetics, transcriptomics, epigenomics and the microbiome, these methodologies provide a valuable complementary approach that broadens the scope of NAMs. This integration enables a deeper understanding of how environmental factors, including bioaerosols, influence respiratory diseases such as asthma and allergic rhinitis, both short-term and long-term impacts, as well as population-level effects of bioaerosols under the influence of climate change and urbanization. For instance, Sadafi et al. (Ref 98) compared the results of a computational fluid dynamics (CFD) model for predicting pharmaceutical aerosol deposition throughout the lungs of asthmatic patients with corresponding in vivo data obtained from single-photon emission computed tomography (SPECT) datasets. They demonstrated that the CFD model can simulate patient-specific deposition when appropriate boundary conditions are applied and can provide information similar to functional imaging tools such as SPECT (Ref 98).

Bioaerosols, such as pollen and fungal spores, interact with environmental pollutants to create dynamic exposure scenarios that are difficult to study using traditional methods alone. Sagona et al. (Ref 99) investigated bioaerosol deposition in the respiratory system using a new bioaerosol-specific lung deposition model (BAIL), compared to two multiple-path models, across three different breathing scenarios and for four bioaerosol species (spores from Aspergillus and Stachybotrys, bacteria and spores from *Bacillus anthracis*). They showed that BAIL predicted higher deposition in the extrathoracic region but lower total deposition for smaller bioaerosols (e.g., bacteria-sized), suggesting a reduced illness risk from deposition in the alveolar region. These results provide a basis for understanding dose–response relationships and bioaerosol health impacts using computational modeling (Ref 99).

Foy et al. (Ref 100) in their study Lung Computational Models and the Role of the Small Airways in Asthma, validated the use of forced oscillation technique-derived R5—R20 as a direct measure of small airway narrowing in asthma. Using computational models combined with clinical data, they demonstrated that small airway narrowing significantly impacts asthma control and quality of life, with deterioration observed beyond a 40% narrowing threshold. The study further predicted that type-2 targeting biologics could reverse small airway narrowing by approximately 40%, improving asthma outcomes (Ref 100).

Under the scenario of increased and structurally altered bioaerosol exposure due to climate and environmental changes, the 2021 study by Atzeni et al., titled “Computational Fluid Dynamic Models as Tools to Predict Aerosol Distribution in Tracheobronchial Airways,” simulated and visualised aerosol trajectories (3–7–10–25 μm) down to the sixth generation of bronchi under steady and dynamic breathing conditions, providing a significant contribution to future research in this field. They created a computational fluid dynamic 3D model of human airways, in order to investigate therapeutic airflow dynamics and aerosol deposition, including the transient behavior in a steady and dynamic breathing physiologically condition. They observed the importance of including anatomic details, such as the curvature of the upper airways, bronchi, and their branching angles, on trajectories and deposition results. This computational modelling enables precise aerosol deposition predictions, optimised medical device designs and improved inhalation protocols for diverse populations (Ref 101).

The increasing number of computational modelling approaches suggests that, as in other fields of medicine, they will resolve layers of complexity and enhance predictability in research on chronic airway diseases and allergies. This approach allows researchers to investigate how dynamic environmental factors influence the respiratory system across multiple scales, from molecular mechanisms to population health.

## Climate Change - NAMs interplay, challenges and limitations of NAMs

NAMs face significant challenges, including scalability, the need for technical expertise and limitations in replicating complex systemic interactions. These constraints are particularly pertinent when attempting to simulate multifaceted environmental conditions induced by climate change, such as elevated CO₂ levels and temperature variations. Addressing these limitations requires advancements in models like Organ-on-a-Chip (OoC) systems, which have shown potential in bridging these gaps.

For instance, Izadifar et al. (Ref 102) introduced a two-channel microfluidic OoC device that enables the recreation of physiologically relevant tissue-tissue interfaces, along with continuous, non-invasive monitoring of key parameters such as transepithelial electrical resistance, oxygen concentration, and pH. This study demonstrates the capacity of OoC systems to replicate dynamic environmental conditions, such as oxygen gradients and hypercapnia, which are critical for understanding respiratory disease mechanisms in scenarios linked to climate change (Ref 102).

NAMs, particularly ALI models, further enhance these capabilities by offering platforms for investigating the interplay between elevated temperatures, increased CO₂ levels and bioaerosol exposure. ALI systems allow for the direct interaction of airborne particles with airway epithelial cells under physiologically relevant conditions, enabling researchers to assess changes in airway inflammation, epithelial barrier integrity and bioaerosol allergenicity in response to altered environmental variables. These models provide critical insights into how climate-induced factors exacerbate respiratory diseases such as asthma and allergic rhinitis.

Additionally, 3D organoids and patient-specific tissue-derived systems offer valuable tools for studying chronic airway diseases. However, limitations such as reproducibility, restricted tissue accessibility and the inability to fully replicate physiological microenvironments remain challenges. Human ASC-derived organoids have demonstrated potential by mimicking patient-specific disease phenotypes, yet their reliance on prior knowledge of culture conditions and accessibility to high-quality tissue samples restricts their broader applicability. These limitations underscore the need for further advancements in model refinement and validation to enhance their translational relevance.

Through advancements like sensor-integrated OoC technologies, NAMs are evolving to address these challenges, offering scalable and versatile platforms for preclinical research. By enabling the simulation of variable environmental conditions, these models improve our understanding of respiratory disease mechanisms under climate change, while also providing robust tools for high-throughput drug screening and regulatory safety testing. Collectively, these approaches underline the transformative potential of NAMs in respiratory research and highlight their growing role in addressing the pressing challenges posed by environmental and climate-related changes.

## Future directions and conclusion

The development of novel approach methodologies (NAMs) has significantly advanced the study of airway diseases such as asthma and allergic rhinitis, which are most closely related to climate change, environmental factors and inhaled bioaerosols. These models, including lung organoids, PCLS, ALI cultures and lung-on-a-chip systems, provide a more accurate representation of human airway function, offering better simulation of environmental factors like allergens and pollutants. They help us understand how these factors exacerbate asthma and allergic diseases at the cellular and tissue levels. However, challenges remain in replicating complex environmental conditions, such as elevated CO₂ levels and temperature fluctuations induced by climate change, which require further advancements in models like Organ-on-a-Chip (OoC) systems (Ref 102).

NAMs offer substantial promise for improving the translation of preclinical findings to human clinical outcomes, particularly in studying bioaerosols and their effects on asthma and allergies. The ability of these models to simulate dynamic lung environments and monitor key parameters in real time is essential for understanding respiratory disease mechanisms, especially in the context of climate change and urbanization. Despite their potential, NAMs still face limitations such as scalability, the need for technical expertise and challenges in reproducing complex systemic interactions.

The integration of computational modeling with novel approach methodologies (NAMs), such as lung-on-a-chip models, air–liquid interface (ALI) cultures and precision-cut lung slices (PCLS), play a key role in accurately simulating complex bioaerosol exposures in lung airways. This approach enhances our understanding of how environmental factors, including pollutants and bioaerosols, influence respiratory diseases like asthma and allergic rhinitis. Computational models help predict aerosol deposition and the effects of these exposures, as seen in studies like those by Sadafi et al. (Ref 98), which demonstrated how CFD models can simulate patient-specific aerosol deposition. Additionally, integrating data from genetics, epigenomics and the microbiome with NAMs provides a more comprehensive view of disease mechanisms. This combined approach offers crucial insights into both short-term and long-term impacts of bioaerosol exposure, especially under changing environmental conditions such as climate change and urbanization. By simulating dynamic interactions between bioaerosols and the respiratory system, these models support the development of more targeted therapies and better regulatory measures for managing respiratory diseases.

Ultimately, the widespread adoption of NAMs across laboratories will help address the pressing health challenges posed by climate change, enabling us to gain deeper insights into the effects of environmental stressors on lung function and disease progression. The transformative potential of these models in respiratory research highlights their growing role in shaping the future of environmental health science. We foresee that the limitations in simulating complex systemic interactions, including scalability issues and the need for specialized technical expertise, will be addressed by an increasing number of expert centers.
